# Validity of Resting Energy Expenditure Predictive Equations before and after an Energy-Restricted Diet Intervention in Obese Women

**DOI:** 10.1371/journal.pone.0023759

**Published:** 2011-09-06

**Authors:** Jonatan R. Ruiz, Francisco B. Ortega, Gerardo Rodríguez, Pilar Alkorta, Idoia Labayen

**Affiliations:** 1 Department of Physical Education and Sport, School of Physical Activity and Sport Sciences, University of Granada, Granada, Spain; 2 Unit for Preventive Nutrition, Department of Biosciences and Nutrition at NOVUM, Karolinska Institutet, Huddinge, Sweden; 3 Department of Medical Physiology, School of Medicine, University of Granada, Granada, Spain; 4 Department of Pediatrics, University of Zaragoza, Zaragoza, Spain; 5 Department of Nuclear Medicine, Hospital of Santiago Apóstol, Vitoria, Spain; 6 Department of Nutrition and Food Science, University of the Basque Country, Vitoria, Spain; Institut Pluridisciplinaire Hubert Curien, France

## Abstract

**Background:**

We investigated the validity of REE predictive equations before and after 12-week energy-restricted diet intervention in Spanish obese (30 kg/m^2^>BMI<40 kg/m^2^) women.

**Methods:**

We measured REE (indirect calorimetry), body weight, height, and fat mass (FM) and fat free mass (FFM, dual X-ray absorptiometry) in 86 obese Caucasian premenopausal women aged 36.7±7.2 y, before and after (n = 78 women) the intervention. We investigated the accuracy of ten REE predictive equations using weight, height, age, FFM and FM.

**Results:**

At baseline, the most accurate equation was the Mifflin *et al.* (Am J Clin Nutr 1990; 51: 241–247) when using weight (bias:−0.2%, P = 0.982), 74% of accurate predictions. This level of accuracy was not reached after the diet intervention (24% accurate prediction). After the intervention, the lowest bias was found with the Owen et al. (Am J Clin Nutr 1986; 44: 1–19) equation when using weight (bias:−1.7%, *P* = 0.044), 81% accurate prediction, yet it provided 53% accurate predictions at baseline.

**Conclusions:**

There is a wide variation in the accuracy of REE predictive equations before and after weight loss in non-morbid obese women. The results acquire especial relevance in the context of the challenging weight regain phenomenon for the overweight/obese population.

## Introduction

The largest component of daily energy expenditure, especially in people with sedentary lifestyle, is resting energy expenditure (REE). To determine reliable REE measurements in obese individuals is important in order to establish reachable goals for dietary intervention and weight loss [1]. REE can be objectively and accurately measured through indirect calorimetry; however, their use is limited in most dietetic settings due to their high cost, the need of qualified and trained technicians and time constraints [2]. Hence, REE estimation by mathematical equations developed from direct or indirect calorimetry was frequently adopted as the major alternative method.

The validity of REE predictive equations is under debate, especially in obese individuals [3]. Indeed, it is likely that the inaccuracy of REE predictive equations in obese subjects might be one of the reasons explaining the low efficacy of low caloric diet treatments [4], [5]. REE highly depends on body size and body composition, but considerable variability exists among individuals after taking into account several key variables such as age, sex, weight, height, fat free mass (FFM), fat mass (FM), sex-hormonal status or ethnicity [6], [7], [8], [9], [10]. Differences in the level of overweight/obesity, as well as the participants' age range are also potential factors explaining the accuracy/inaccuracy of the REE predictive equations and the differences observed in the few available studies [11], [12], [13], [14].

Several studies have assessed the validity of REE predictive equations in overweight and obese subjects (body mass index (BMI)>25 kg/m^2^) [3], [15], [16], and in morbid obese people (BMI>40 kg/m^2^) [17], [18]. Less studies have however examined the validity of REE predictive equations specifically in non-morbid obese women (30 kg/m^2^>BMI<40 kg/m^2^) [3], [15], [19]. All these studies included women with a wide age range and examined pre- and post-menopausal women together despite previous reports showed that REE decreases with aging and may also decrease in women as a result of the menopause [20].

Evidence exists that weight loss leads to a reduction in REE beyond that explained by the decrease in FFM and FM. This phenomenon has been described as “*adaptive thermogenesis*” or “*metabolic adaptation*” [21], [22]. Metabolic adaptation occurs when the body countervails energy restriction by decreasing REE [23]. This decrease is beyond the expected decrease in REE due to changes in body weight, which could account in part for the common cessation of weight loss observed after 12–20 weeks of energy restriction [24]. Our understanding of the molecular mechanisms that regulate energy homeostasis has increased remarkably over the past decade, however, little is known about the effect of energy restriction on adaptive changes in energy homeostasis. Moreover, the accuracy of REE predictive equations after a weight loss program and the consequent “*metabolic adaptation*” has not been thoroughly examined [12] despite its implication in the weight regulation after a dietary intervention and the challenging weight regain phenomenon in the overweight/obese population. Indeed, a variety of factors are known to influence weight maintenance in overweight and obese persons [25]. To better understand the accuracy of REE predictive equations in obese persons right after an energy intervention program may help to patients to prevent weight regain.

In the present study, we systemically searched for REE predictive equations including (or not) body composition measurements and compared the estimated vs. the measured REE before and after a 12-week energy-restricted intervention. Therefore, the purpose of this study was to investigate the validity of REE predictive equations before and after a 12-week energy-restricted diet intervention in Spanish obese, non-morbid pre-menopausal Caucasian women.

## Methods

### Participants

Participants were obese (BMI inclusion criteria: 30–39.9 kg/m^2^) pre-menopausal Caucasian women from Vitoria (North Spain), aged between 19 and 49 years, non-physical active (<20 minutes on <3 days/week), and with stable weight (body weight changes <3 kg) over the last 3 months. None of them were enrolled in a weight loss program. Each participant underwent a comprehensive medical examination and laboratory tests for blood glucose, plasma proteins, red and white blood cells, platelets, and liver, thyroid and kidney function. Exclusion criteria included history of cardiovascular disease or diabetes, pregnancy, total cholesterol levels >300 mg/dL (7.85 mmol/L), levels of triglyceride >300 mg/dL (3.38 mmol/L) and blood pressure >140/90 mmHg. We also excluded women under medication for hypertension, hyperlipidemia, hyperuricemia or other illness. Smoking, or use of oral contraceptives, was not considered an exclusion criterion. All women received verbal and written information about the nature and purpose of the survey, and all of them gave written consent for participation in the study. This study was in accordance with the Helsinki II Declaration and was approved by the Ethical Committee in Hospital of Txagorritxu (Vitoria).

A total of 86 obese women were enrolled in the baseline measurements. Among these participants, 83 voluntary participated in a 12-week energy-restricted diet intervention. Four participants left the study due to inability to follow the research protocol and 1 due to pregnancy (dropout rate = 6%). A total of 78 women participated in the post-intervention measurements. The final sample (n = 78) did not differ in key characteristics at baseline (i.e., age, REE, body weight, BMI or FFM) from the original sample (n = 83, P = 0.676, 0.454, 0.194, 0.406, 0.256, respectively).

### Design

The present study was designed as a 12 weeks controlled weight loss program. Body weight reduction was induced by a low energy mixed (55% carbohydrates, 30% lipids and 15% proteins) diet providing 600 kcal less than individually estimated energy requirements based on measured REE. The individual energy requirements were estimated by indirect calorimetry (ventilated hood system) at baseline and multiplied by a factor of 1.3, as corresponds to a low physical activity level.

Energy content and macronutrient composition of diets were according to the American Diabetes Association nutrition recommendations [26], [27]. Diets were designed to achieve weight losses of 0.5 to 1 kg per week; such diets are considered as a low risk intervention [27], [28]. To optimize compliance, dietary instructions were reinforced weekly by a dietician. The consultation included both nutritional assessment and weighing. A 3-d weighed-food record of 2 weekdays and 1 weekend day was performed before the study and during the last week of intervention. One-day weighed-food records were completed in the week 2, 5 and 7. Low energy diets and dietary records were analyzed by using a food-nutrient database (Alimentación y Salud, BitASDE General Médica Farmacéutica, Albocacer, Valencia, Spain).

The study examinations were performed before and after 12 weeks of dieting in the Unit of Clinic Assays of LEIA Foundation (Txagorritxu Hospital, Vitoria).

### Assessment of resting energy expenditure

Respiratory exchange measurements by indirect calorimetry were used to estimate REE following the recommended measurements conditions [29]. The participants were asked not to perform any intense physical activity the day immediately before the measurement. For each examination day (at baseline and 12 weeks after), participants arrived by car or bus at the Hospital at 8–9 a.m. in a fasting condition of at least 12 hours. The measurements were taken in peaceful and relaxing environment and at a constant temperature (∼24°C) and humidity (∼50%). Women were in a supine position and awake. After 30 min of rest, respiratory exchange measurements were determined by means of an open-circuit computerised indirect calorimeter (Vmax, Sensormedics, Germany) using a transparent, ventilated canopy-hood system and after daily calibration with a reference gas mixture (95% O_2_, 5% CO_2_). The first and final 5 min of each set were routinely discarded and the mean value of the remaining 20 min was used for the calculations, once the steady-state conditions were obtained. The coefficient of variation (CV) was <10%. If steady-state could not be maintained that long, a 10-min segment with CV<5% was accepted. This instrument has shown to be valid to assess REE and respiratory exchange ratio [30]. Urine was collected in the postabsorptive state to determine nitrogen output. REE was calculated from O_2_ and CO_2_ volumes, as well as from urine excretion nitrogen values, by using the formula of Weir and expressed as kcal/day as reported elsewhere [31], [32], [33].

### Body composition

Body weight (±10 g) was measured after voiding using a digital integrating scale (SECA 760). Height was measured to the nearest 5 mm using a stadiometer (SECA 220) at the start of the study. Body mass index (BMI) was calculated as weight (kg)/height (m)^2^. Dual Energy X-ray Absorptiometry (DXA) measurements were performed within ±3 days of the pre- and post-intervention examinations. A DXA scanner 140 (HOLOGIC, QDR 4500W) with QDR software for windows version 12.4 was used to estimate fat mass (FM) and FFM. All DXA scans, which were completed with the same device and software, were performed by the same qualified technician who was trained in the operation of the scanner, the positioning of subjects, and the analysis of results according to manufacturer's guidelines and adhering to accepted methodology.

### Resting energy expenditure predictive equations

Predictive equations were obtained by screening previous publications. We selected REE predictive equations based on the following criteria: (i) equations based on body weight, height, sex, and/or FM and FFM; and (ii) developed in adults. Exclusion criteria included: (i) equations derived only from elderly populations, patients or athletes; (ii) small (n<20%) proportion of overweight; (iii) small sample size (n<50); and (iv) specific ethnic groups or insufficient information. According to these criteria, we included a total of 10 REE predictive equations [19], [34], [35], [36], [37], [38] (**Table 1**).

**Table 1:** Resting energy expenditure predictive equations.

Reference	REE predictive equations
HB1919 [34]	Weight (kg)×9.5634+Height (cm)×1.8496−Age (y)×4.6756+655.0955
Owen et al. [35] Weight	Weight (kg)×7.18+795
Owen et al. [35] Fat free mass	19.7×Fat free mass (kg)+334
Mifflin et al. [36] Weight	9.99×Weight (kg)+6.25×Height (cm)−4.92×Age (y)+166×Sex−161
Mifflin et al. [36] Fat free mass	19.7×Fat free mass (kg)+413
FAO/OMS/UNU [37] Weight	Age 18–30 y: 14.7×Weight (kg)+496
	Age 30–60 y: 8.7×Weight (kg)+829
FAO/OMS/UNU, [37] Weight and Height	Age 18–30 y: 13.3×Weight (kg)+334×Height (m)+35
	Age 30–60: 8.7×Weight (kg)−25×Height (m)+865
Weijs & Vansant [19] Weight and Height	Weight (kg)×14.038+Height (cm)×4.498−Age (y)×0.977−221.631
Bernstein et al. [38] Weight	7.48×Weight (kg)−0.42×Height (cm)−3×Age (y)+844
Bernstein et al. [38] Fat free mass, Fat mass	19.02×Fat free mass+3.72×Fat mass−1.55×Age (y)+236.7

### Statistical analysis

We conducted paired t-tests to analyze differences in changes on body weight, BMI, FM and FFM after a 12-week energy-restricted diet intervention, and so we did to analyze the bias (mean percentage error differences between REE estimations by predictive equations and measured REE values by calorimetry). We calculated the root mean sum of squared errors (RMSE). We considered an accurate estimation when the equation predicted between 90% and 110% of the measured REE, such as in previous studies [3], [15], [19], whereas we classified as under-prediction and over-prediction when the estimation was <90% and >110% of the measured REE, respectively. The percentage of women that had a predicted REE within ±10% of the measured REE was considered as an index of accuracy at individual level. Nevertheless, as this range could be considered too wide in the clinical practice [4], we included also the percentage of accurate predictions within ±5% of the measured REE.

The agreement between REE predicted equations and measured REE was graphically examined [39] by plotting the difference between the predicted and the measured REE against the measured REE [40]. We calculated the mean difference, 95% confidence intervals of the difference, and the 95% limits of agreement (mean difference ±1.96SD of the difference). The heteroscedasticity, that is, the association between the magnitude of the measurement (i.e. measured REE) and the difference between the predicted and measured REE, was examined by regression analysis, entering the absolute difference between the predicted and measured REE as dependent variable and the measured value as independent variable. Data were analyzed by using PASW (Predictive Analytics SoftWare, v. 18.0 SPSS Inc., Chicago, IL, USA).

## Results

The characteristics of the study sample at baseline and after a 12-week energy-restricted diet intervention are shown in **Table 2**. Women had lower BMI, FM and FFM after the intervention (all *P*<0.001).

**Table 2:** Descriptive characteristics of the study participants before (baseline) and after a 12-week energy-restricted diet intervention.

	Baseline(n = 86)		Post 12-week diet intervention (n = 78)	
	*mean*	*sd*	*mean*	*sd*
Age (y)	36.6	7.2		
Weight (kg)	89.5	10.2	81.2	10.0
BMI (kg/m^2^)*	33.9	2.8	30.7	2.8
Fat mass (kg)	37.8	6.3	32.6	6.2
Fat free mass (kg)	50.6	5.4	47.8	5.3

*BMI, body mass index; sd, standard deviation.

**Table 3** shows (before and after 12-week diet intervention) the means and SD values of measured REE and estimated REE with the selected predictive equations, the percentage bias, the maximum values observed for negative errors (under-prediction) and positive errors (over-prediction), the RMSE (in kcal/d), and the percentage of under and over predictions. At baseline, we observed a significant bias in all the REE predictive equations (all *P*<0.05) except in the equation reported by Mifflin et al. [36] when using weight (bias: −0.2%, *P* = 0.982), RMSE of 136 kcal/d, 74% of accurate predictions, 14% under-predictions and 12% over-predictions. The highest bias observed corresponded to the equation reported by Bernstein et al. [38] when including FFM and FM (−25%, *P*<0.001), RMSE of 341 kcal/d, 5% accurate predictions and 95% under predictions.

**Table 3:** Evaluation of resting energy expenditure (REE) predictive equations in Spanish obese women before (n = 86) and after a 12-week energy-restricted diet intervention (n = 78) based on bias, root mean squared error (RMSE), and percentage accurate prediction.

REE predictive equation	REE*	SD	Bias†	Maximum negative error‡	Maximum positive error§	RMSE	Accurate predictions∥	Accurate predictions¶	Under predictions**	Over predictions††
*Baseline (n = 86)*	*kcal/d*		*%*	*%*	*%*	*kcal/d*	*%*	*%*	*%*	*%*
RMR measured	1564	172								
HB 1919 [34]	1640	115	4.6	−20	22	152	41	66	6	28
Owen et al. [35] Weight	1438	73	−8.7	−32	9	185	23	53	56	0
Owen et al. [35] Fat free mass	1331	107	−17.3	−41	5	259	9	23	77	0
Mifflin et al. [36] Weight	1565	141	−0.2	−31	18	136	38	74	14	12
Mifflin et al. [36] Fat free mass	1410	107	−10.7	−33	10	192	24	40	59	1
FAO/OMS/UNU, [37] Weight	1661	154	5.5	−18	26	182	37	58	6	36
FAO/OMS/UNU, [37] Weight and Height	1366	267	−17.7	−55	24	326	5	16	71	13
Weijs & Vansant, [19] Weight and Height	1726	163	9.1	−19	24	213	29	52	1	47
Berstein et al. [38] Weight and Height	1336	79	−17.1	−43	4	266	13	24	76	0
Berstein et al. [38] Fat free mass, Fat mass	1284	115	−21.9	−52	−1	302	3	8	92	0

*Post 12-week diet (n = 78)*	*kcal/d*		*%*	*%*	*%*	*kcal/d*	*%*	*%*	*%*	*%*
RMR measured	1403	144								
HB 1919 [34]	1559	114	10.0	−8	26	189	21	56	0	44
Owen et al. [35] Weight	1378	72	−1.7	−19	17	111	44	81	12	7
Owen et al. [35] Fat free mass	1276	105	−10.0	−30	10	156	23	45	54	1
Mifflin et al. [36] Weight	1641	141	14.4	−6	29	261	4	24	0	76
Mifflin et al. [36] Fat free mass	1355	105	−3.5	−21	15	103	38	82	14	4
FAO/OMS/UNU, [37] Weight	1566	127	10.3	−6	25	204	23	51	0	49
FAO/OMS/UNU, [37] Weight and Height	1556	119	9.7	−6	25	194	26	54	0	56
Weijs & Vansant, [19] Weight and Height	1610	163	12.6	−12	27	238	10	32	1	67
Berstein et al. [38] Weight and Height	1170	112	−20.1	−45	−2	250	24	13	87	0
Berstein et al. [38] Fat free mass, Fat mass	1211	114	−16	−40	1	212	8	24	76	0

*As measured.

† Mean percentage error between predictive equation and measured value.

‡ The largest underprediction observed with this predictive equation as a percentage of the measured value.

§ The largest overprediction observed with this predictive equation as a percentage of the measured value.

∥ Percentage of women predicted by this predictive equation within 5% of the measured value.

¶ Percentage of women predicted by this predictive equation within 10% of the measured value.

** Percentage of women predicted by this predictive equation <10% of the measured value.

†† Percentage of women predicted by this predictive equation >10% of the measured value.

After 12-week energy-restricted diet intervention, the equation reported by Owen et al. [35] when using weight was the one with the lowest bias (−1.7%, *P* = 0.044), RMSE of 106 kcal/d, 81% accurate prediction, 12% under-predictions and 7% over-predictions. The equation reported by Mifflin et al. [36] when using weight had a bias of 14.4% (*P*<0.001), RMSE of 249 kcal/d, 24% accurate predictions and 76% over-predictions. Moreover, the equation reported by Mifflin et al. [36] when using FFM had a bias of −3.5%, RMSE of 103 kcal/d, 82% accurate predictions, 14% under-predictions and 4% over-predictions. The highest bias observed corresponded to the equation reported by Bernstein et al. [38] when including weight and height (−20.1%, *P*<0.001), RMSE of 238 kcal/d, 13% accurate predictions and 87% under-predictions.

**Figure 1:**
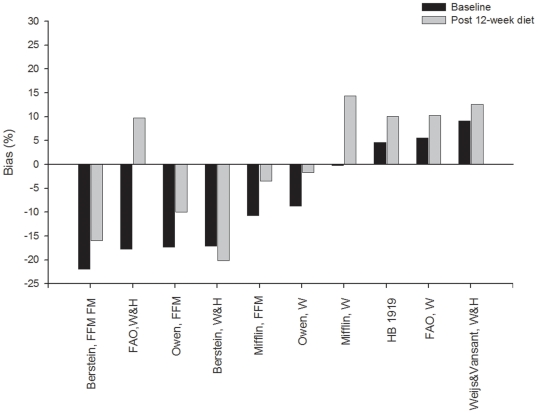
Percentage bias for 10 resting energy predictive equations in Spanish obese women before (baseline, n = 78) and after a 12-week energy-restricted diet intervention (post 12-week diet intervention, n = 78). Data are sorted by mean values at baseline. FFM indicates fat free mass; FM, fat mass; W, weight; W&H, weight and height.

**Figure 2:**
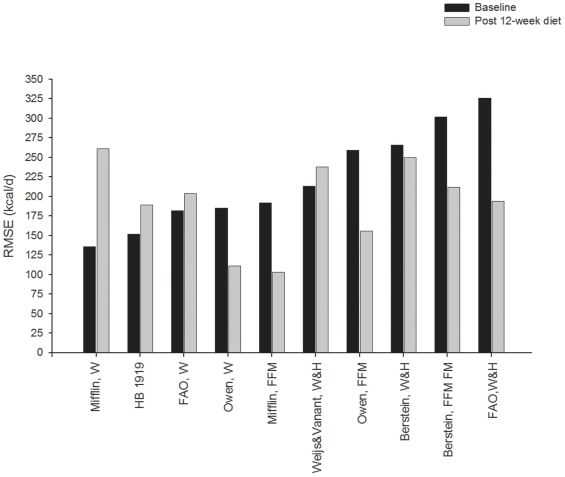
Root mean squared error for 10 resting energy predictive equations in Spanish obese women before (baseline, n = 78) and after a 12-week energy-restricted diet intervention (post 12-week diet intervention, n = 78). Data are sorted by mean values at baseline. FFM indicates fat free mass; FM, fat mass; W, weight; W&H, weight and height.

Percentage bias and RMSE at baseline and after the 12-week energy-restricted diet intervention of those women who completed the intervention (n = 78) is depicted in **Figure 1** and **2**, respectively. Both percentage bias and RMSE at baseline were significantly different compared to that observed after the 12-week diet intervention in all the studied REE predictive equations (all *P*<0.001). The bias observed with the equation reported by Mifflin et al. [36] changed from −0.35% at baseline to 14.4% after 12-week diet intervention (RMSE: 136 and 249 kcal/d, at baseline and after 12-week diet intervention, respectively). Similarly, the bias observed with the equation reported by Owen et al. [35] changed from −8.7% at baseline to −1.7% after 12-week diet intervention (RMSE: 185 and 106 kcal/d, at baseline and after 12-week diet intervention, respectively). The HB1919 equation [34] had a predicted REE within ±10% of the measured REE at both baseline (bias 4.6%, RMSE of 152 kcal/d, 66% accurate predictions, 6% under-predictions and 28% over-predictions) and post 12-week diet intervention (bias 10.0%, RMSE of 180 kcal/d, 56% accurate predictions and 44% over-predictions).

**Figure 3:**
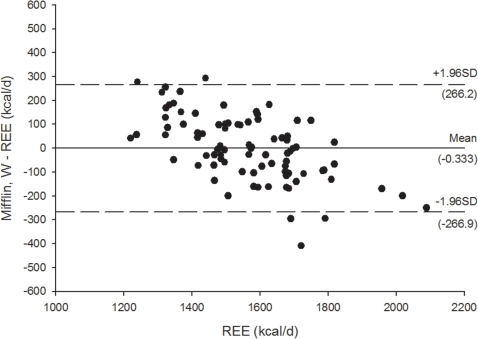
Bland Altman plots for the Mifflin et al. (34) for resting energy expenditure predictive equations in Spanish obese women before a 12-week energy-restricted diet intervention (baseline, n = 78). Solid line represents the mean difference (bias) between predicted and measured resting energy expenditure (REE). Upper and lower dashed lines represent the 95% limits of agreement (mean difference ±1.96 SD of the difference).

**Figure 3** **and** **4** shows the Bland Altman plots for the Mifflin et al. [36] REE predictive equations, **Figure 5** **and** **6** shows the Bland Altman plots for the Owen et al. [35] REE predictive equations, and **Figure 7** **and** **8** shows the Bland Altman plots for the HB1919 [34] REE predictive equations. We observed heteroscedasticity in all equations (all *P*<0.001). There was an inverse association between the magnitude of the measurement (i.e. measured REE) and the difference of the predicted and measured REE.

**Figure 4:**
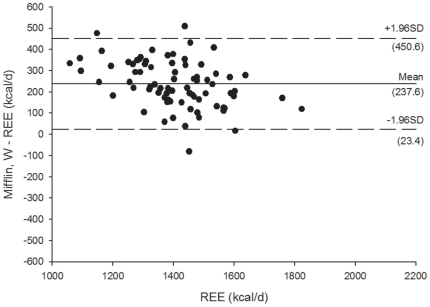
Bland Altman plots for the Mifflin et al. (34) (weight) for resting energy expenditure predictive equations in Spanish obese women after a 12-week energy-restricted diet intervention (post 12-week diet intervention, n = 78). Solid line represents the mean difference (bias) between predicted and measured resting energy expenditure (REE). Upper and lower dashed lines represent the 95% limits of agreement (mean difference ±1.96 SD of the difference).

**Figure 5:**
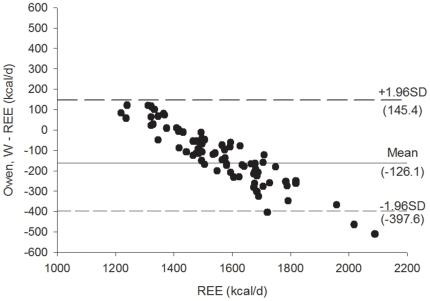
Bland Altman plots for the Owen et al. (33) (weight) for resting energy expenditure predictive equations in Spanish obese women before a 12-week energy-restricted diet intervention (baseline, n = 78). Solid line represents the mean difference (bias) between predicted and measured resting energy expenditure (REE). Upper and lower dashed lines represent the 95% limits of agreement (mean difference ±1.96 SD of the difference).

**Figure 6:**
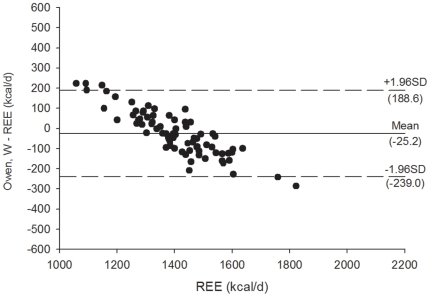
Bland Altman plots for the Owen et al. (33) (weight) for resting energy expenditure predictive equations in Spanish obese women after a 12-week energy-restricted diet intervention (post 12-week diet intervention, n = 78). Solid line represents the mean difference (bias) between predicted and measured resting energy expenditure (REE). Upper and lower dashed lines represent the 95% limits of agreement (mean difference ±1.96 SD of the difference).

**Figure 7:**
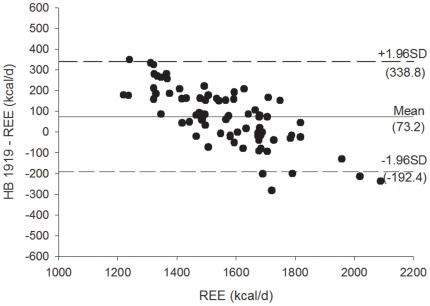
Bland Altman plots for the HB 1919 (32) for resting energy expenditure predictive equations in Spanish obese women before a 12-week energy-restricted diet intervention (baseline, n = 78). Solid line represents the mean difference (bias) between predicted and measured resting energy expenditure (REE). Upper and lower dashed lines represent the 95% limits of agreement (mean difference ±1.96 SD of the difference).

**Figure 8:**
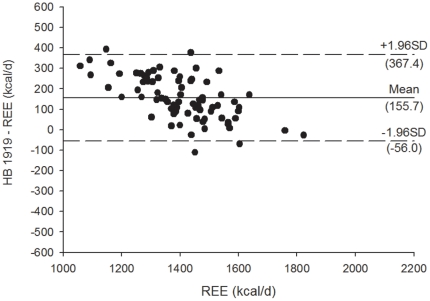
Bland Altman plots for the HB 1919 (32) for resting energy expenditure predictive equations in Spanish obese women after a 12-week energy-restricted diet intervention (post 12-week diet intervention, n = 78). Solid line represents the mean difference (bias) between predicted and measured resting energy expenditure (REE). Upper and lower dashed lines represent the 95% limits of agreement (mean difference ±1.96 SD of the difference).

## Discussion

The results of this study indicate that the best equation to estimate REE before a weight loss program in obese (BMI: 30–39.9 kg/m^2^) pre-menopausal Caucasian women is the equation reported by Mifflin *et al.* [36] when using weight, whereas the Owen *et al.* [35] equation (when using weight) is the best to estimate REE after a 12-week energy-restricted diet intervention in the same women. The Mifflin equation provides 74% accurate predictions before the diet intervention, yet, this level of accuracy cannot be reached after the 12-week diet intervention (24% accurate prediction). The Owen equation provides 81% accurate predictions after a 12-week energy-restricted diet intervention, although it only provides 53% accurate predictions at baseline. The average weight loss percentage in this study was close to 10%, which matches well the current clinical recommendations for overweight or obese persons [41], [42]. These findings are clinically relevant and suggest that the best equation to estimate REE in Caucasian obese women greatly depends on whether the patient has recently participated or not on an energy-restricted diet intervention.

The Mifflin equation was derived from a sample of 498 men and women, which included non-overweight, overweight and obese subjects and whose age ranged between 19 and 78 years. Several studies proposed this equation as the most valid to estimate REE in non-obese subjects aged 18 to 78 years (82% accurate predictions) [3], and in overweight and obese subjects aged 19 to 69 years (78% accurate predictions). There is some support also for using the Mifflin equation in European American females [43] and extremely obese females [17]. Likewise, Frankenfield et al. [14] in a validation study conducted in a cohort of 20 adults (12 women) aged 18 to 69 years, observed that 70% were within the range of agreement (±10% of measured REE). Weijs [15] tested the accuracy of 27 REE predictive equations in Dutch and U.S. overweight (BMI between 25 and 40 kg/m^2^) adults (18–65 years) and reported that the Mifflin equation provided almost 80% accurate predictions for U.S. adults and performed well across sex and BMI groups; but, for the whole sample of Dutch adults, none of the equations reached this level of accuracy. Nevertheless, the Mifflin equation provided 68% of accurate predictions for obese Dutch women (n = 74 women aged <65 years) from this same sample. More recently, Weijs et al. [19] examined the validity of REE predictive equations in 536 normal weight to morbid obese Belgian women and showed that either the original Harris–Benedict (69% of accurate predictions) or the Mifflin equation (68% of accurate predictions) can be used with accuracy to predict REE across a wide range of body weight (BMI, 18.5–50 kg/m^2^). However, they noticed that the accuracy of both Harris–Benedict and the Mifflin equations was fairly low when considering the BMI range of 30–40 kg/m^2^. Our results confirm and extend these findings, and provide more evidence for the use of the Mifflin equation in pre-menopausal, non-morbid obese women with weight stability of at least 3 months.

The observed variability in the accuracy of the Mifflin equation for predicting REE in obese women in the above mentioned studies could be explained by (i) the inclusion of both pre-menopausal and post-menopausal women; and (ii) the lack of control about weight stability during the previous months. REE decreases beyond values expected from body weight and body composition loses as result of energy restriction [24]. Thus, this metabolic adaptation could affect the validity of the equations which are derived from data of individuals with a stable energy balance. Interestingly, we observed that the error of all equations changed after losing ∼9% of body weight and that we cannot use with accuracy the same equation before and after a weight loss program. Indeed, both percentage bias and RMSE at baseline were significantly different compared to those observed after the 12-week diet intervention in all the studied REE predictive equations. Moreover, the percentage of over-predictions increased after weight loss in all the predictive equations. Siervo et al. [12] explored the influence of losing at least 5% of body weight in the accuracy of REE predictive equations in 31 subjects, and reported that the Owen equation was the most accurate, which is in agreement with our own findings. However, no information regarding the energy restriction treatment (diet composition, duration of intervention, etc.) or study sample characteristics (i.e. sex, age and BMI range) was provided, which hamper further between study comparisons. Interestingly, the Mifflin equation using FFM after the 12-week diet intervention had similar accuracy than the Owen equation. As far as we are aware, ours is the first study examining the influence of an energy restriction treatment on the validity of REE predictive equations in a well characterized sample of obese pre-menopausal Caucasian women. Whether the best equation to estimate REE after an energy-restricted diet intervention varies depending on the length of the intervention is not known. Moreover, the possible variation in the validity of REE equations when weight loss is achieved through programs combining hypocaloric diet and exercise remains to be elucidated. Future studies should address these issues. Furthermore, future studies should also investigate which is the best REE predictive equation after a follow up period of, for instance, 3–6 months in women that followed an energy-restricted diet intervention.

The Harris-Benedict equation is one of the most commonly used in clinical practice and, as it is the oldest, has undergone the most extensive validation. An expert panel evaluated 25 of validation studies and showed that accurate REE predictions occurred in 45% to 80% of individuals and that REE overestimates occurred more frequently than underestimates [3]. We observed that the Harris-Benedict equation had 66% of accurate prediction (6% under-predictions and 28% over-predictions) with a bias of 4.6% at baseline, and 56% of accurate prediction (0% under-predictions and 44% over-predictions) with a bias of 10.0% after 12-week of energy-restricted diet intervention. It has been reported that this equation systematically overestimates REE by approximately 5% [12], [14], [36], [44], which is in agreement with our results at baseline. In the current study, we extend these observations to the over-estimation of REE by the Harris-Benedict equation after 12-week of energy-restricted diet that reached the 10% of bias in obese women (RMSE of 180 kcal/d and 44% over-predictions).

Previous studies noted that the error in the prediction of REE was more likely in obese than in non-obese individuals [14], [19]. In our study, we observed that there was an inverse association between the magnitude of the measurement (i.e. measured REE) and the difference between the predicted and the measured REE. Indeed, the error in the REE estimation increased when increasing the magnitude of REE, which is closely associated with body size. In consequence, the use of adjusted body weight in the prediction of REE for the calculation of energy content in the prescription of low caloric diets does not seem a reasonable maneuver in clinical practice.

In agreement with other studies [14], [15], [19], we noted that the inclusion of body composition (FFM and/or FM) did not improve the accuracy of REE prediction while women were within a stable weight period. Likewise, the most accurate predictive equation after the 12-week diet intervention program, the Owen equation, does not consider body composition. This is a relevant finding because weight derived equations are more feasible in clinical practice. Only the Mifflin et al. equation that includes FFM was enough accurate in predicting REE after weight loss in obese pre-menopausal women.

Although this study has strengths, we acknowledge several limitations. We did not measure sex hormones level to ensure that women were at the same phase of the menstrual cycle at baseline and after the dietary treatment. However, it is reasonable to think that they were at almost the same phase; indeed we designed the length of the treatment to be multiple of 4 weeks. Second, we did not exclude participants if they smoked, yet, smoking was not allowed before performing the indirect calorimetry measurement [29]. In our study conditions of measurement were strictly controlled and standardized, which is certainly a strength. Moreover, it is worth mentioning that our sample was more homogeneous than other previously reported due to the strict inclusion criteria and to the highly controlled intervention. Women were Spanish Caucasian, non-diabetic and non-morbid obese pre-menopausal women and followed an energy-restricted diet with similar macronutrient composition based on Mediterranean dietary habits, and whose energy content was estimated from measured REE. The use of DXA to measure body composition before and after weight loss should also be acknowledged.

In conclusion, this study shows that there is a wide variation in the accuracy of REE predictive equations before and after weight loss in non-morbid obese women. These findings are clinically relevant and suggest that the best equation to estimate REE greatly depends on whether the patient has recently participated or not on an energy-restricted diet intervention. The results acquire also especial relevance in the context of the challenging weight regain phenomenon for the overweight/obese population. Our findings confirm that the best equation to estimate REE in non-morbid obese, pre-menopausal women is the equation reported by Mifflin et al. [36]. However, the equation that best estimates REE in obese women after a 12-week energy-restricted diet intervention is the equation reported by Owen et al. [35]. There is a need to develop best practices with focus on weight regain [45], and understanding the accuracy of REE predictive equations in obese individuals after an energy intervention program may be such an example. Future studies in post-menopausal women, in morbid obese women, in men, and in other ethnicities are needed to further investigate the validity of REE predictive equations before and after and energy-restricted diet intervention so that the efficacy of weigh loss programs can be improved.
